# Supplementary figures and images for: Identifying topologically associating domains using differential kernels
Source: PLoS Comput Biol. 2024 Jul 15;20(7):e1012221. doi: 10.1371/journal.pcbi.1012221 (PMC11249266; doi:10.1371/journal.pcbi.1012221)

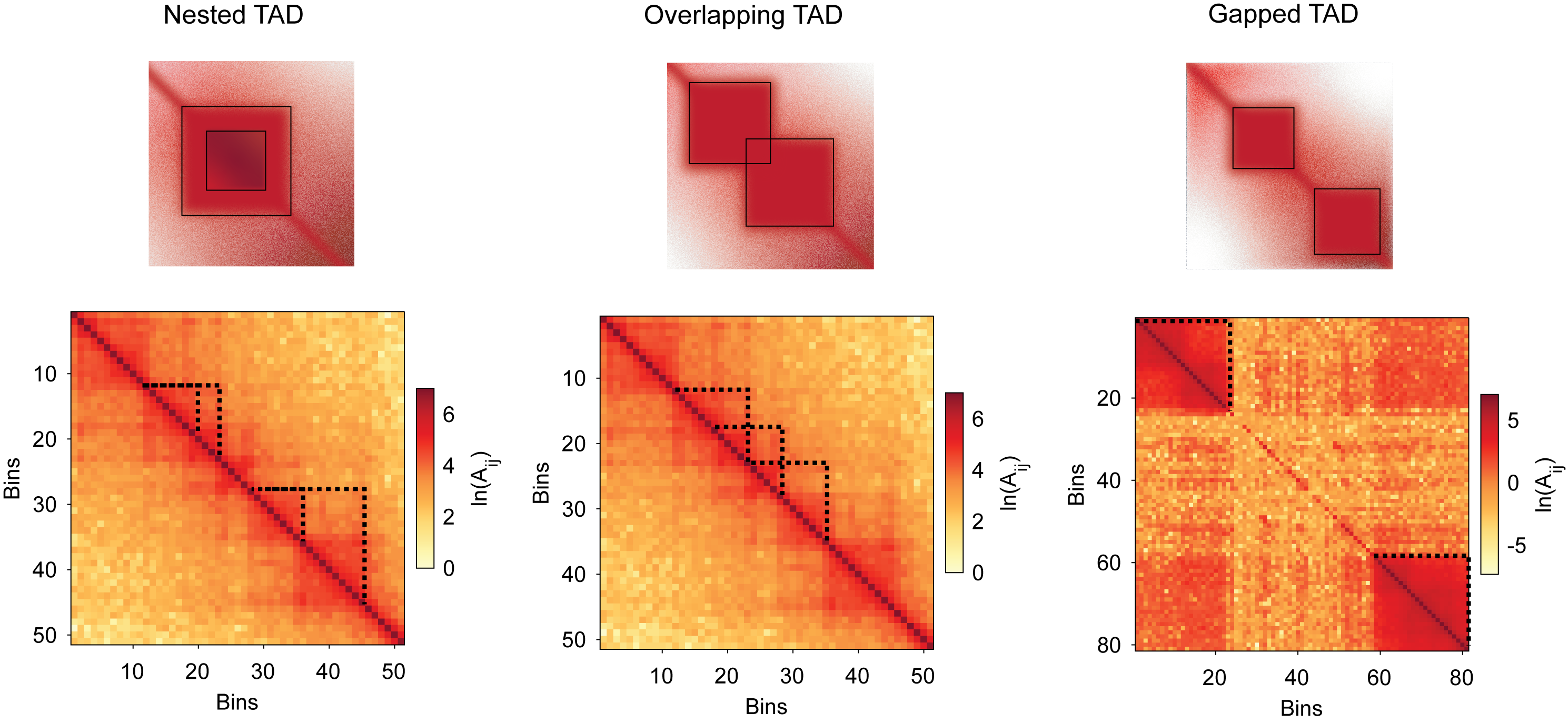

Supplement: S1 Fig — We illustrate graphically (from left to right) nested, overlapping, and gapped TADs in the top row. In the bottom row, below each type of TAD, we show an example of that particular type of TAD (outlined using dotted lines) in an experimental Hi-C map of chromosome 6 from the human GM12878 cell line. (TIF) [file pcbi.1012221.s001.tif]
